# *TaER* Expression Is Associated with Transpiration Efficiency Traits and Yield in Bread Wheat

**DOI:** 10.1371/journal.pone.0128415

**Published:** 2015-06-05

**Authors:** Jiacheng Zheng, Zhiyuan Yang, Pippa J. Madgwick, Elizabete Carmo-Silva, Martin A. J. Parry, Yin-Gang Hu

**Affiliations:** 1 State Key Laboratory of Crop Stress Biology for Arid Areas and College of Agronomy, Northwest A&F University, Yangling, Shaanxi, China; 2 Department of Plant Biology and Crop Science, Rothamsted Research, Harpenden, Hertfordshire, United Kingdom; 3 Institute of Water Saving Agriculture in Arid Regions of China, Yangling, Shaanxi, China; Murdoch University, AUSTRALIA

## Abstract

*ERECTA* encodes a receptor-like kinase and is proposed as a candidate for determining transpiration efficiency of plants. Two genes homologous to *ERECTA* in *Arabidopsis* were identified on chromosomes 6 (*TaER2*) and 7 (*TaER1*) of bread wheat (Triticum aestivum L.), with copies of each gene on the A, B and D genomes of wheat. Similar expression patterns were observed for *TaER1* and *TaER2* with relatively higher expression of *TaER1* in flag leaves of wheat at heading (Z55) and grain-filling (Z73) stages. Significant variations were found in the expression levels of both *TaER1* and *TaER2* in the flag leaves at both growth stages among 48 diverse bread wheat varieties. Based on the expression of *TaER1* and *TaER2*, the 48 wheat varieties could be classified into three groups having high (5 varieties), medium (27 varieties) and low (16 varieties) levels of *TaER* expression. Significant differences were also observed between the three groups varying for *TaER* expression for several transpiration efficiency (TE)- related traits, including stomatal density (SD), transpiration rate, photosynthetic rate (A), instant water use efficiency (WUEi) and carbon isotope discrimination (CID), and yield traits of biomass production plant^-1^ (BYPP) and grain yield plant^-1^ (GYPP). Correlation analysis revealed that the expression of *TaER1* and *TaER2* at the two growth stages was significantly and negatively associated with SD (P<0.01), transpiration rate (P<0.05) and CID (P<0.01), while significantly and positively correlated with flag leaf area (FLA, P<0.01), A (P<0.05), WUEi (P<0.05), BYPP (P<0.01) and GYPP (P<0.01), with stronger correlations for *TaER1* than *TaER2* and at grain-filling stage than at heading stage. These combined results suggested that *TaER* involved in development of transpiration efficiency -related traits and yield in bread wheat, implying a function for TaER in regulating leaf development of bread wheat and contributing to expression of these traits. Moreover, the results indicate that *TaER* could be exploitable for manipulating important agronomical traits in wheat improvement.

## Introduction

In many regions of the world, water deficits impose serious constraint on plant growth and crop productivity. Plant transpiration efficiency (TE) is critical to plant survival and has important implications for both carbon cycling and water balance. Plants have evolved a variety of ways of controlling TE; understanding this control is essential to underpin attempts to improve crop productivity with limited water availability. TE is affected significantly and variably by canopy characteristics and leaf anatomy (i.e. leaf thickness, mesophyll cell size and position, stomatal density) and activity (stomatal conductance). In *Arabidopsis thaliana*, ERECTA (ER) was demonstrated to regulate the development of leaf architecture, and be a major gene contributing to TE, ER was the major contributor to a locus for carbon isotopic discrimination (Δ) and was negatively related to transpiration efficiency [1]. Thus, understanding the ER genotypic variation of leaf traits will be valuable to in attempts to improve TE, photosynthesis and crop productivity.

ER is associated with numerous functions that affect plant development and TE [2]. The *ER* gene was first isolated from *Arabidopsis thaliana* and belongs to the receptor-like kinase family (RLKs) with an N-terminal extracellular domain and C-terminal intracellular kinase that transduces extracellular signals into the cells to control a wide range of physiological responses [3, 4, 5]. The role of *ER* has been examined by both forward and reverse genetic approaches. Mutations to *ER* in *Arabidopsis* conferred decreased TE, but *ER* complementation led to restoration of TE [1]. In transgenic tomato plants, the expression of a truncated ER protein from *Arabidopsis* (atΔKinase), increased the number of stomata per leaf, transpiration and photosynthetic rates [6]. Over-expression in *Arabidopsis* of the *PdERECTA* gene from *Populus nigra* L. (35S:PdERECTA) increased photosynthetic rate, whilst decreasing transpiration rate and thereby increasing water use efficiency (WUEi) [7]. Complete function loss of three *ER*-family genes (*ER*, *ER-LIKE1* (*ERL1*) and *ERL2*) in *Arabidopsis* resulted in the generation of high-density stomatal clusters and a 50–200% increase of the stomatal index [8]. ER appears to play a central role in the epidermal cell differentiation signaling pathway, inhibiting stomatal development and leading to reduced stomatal density and conductance. Therefore, *ER* is a prime candidate gene for studying the natural diversity of TE and photosynthesis in crops.

Wheat is a major cereal crop in the world, and is cultivated in arid and semi-arid regions of the world, where water deficit and other environmental fluctuations limit its growth, development and yield. Since ER has been theorized to play a major role in plant development and TE for a number of species, this study investigates the multi-gene *ER* family in bread wheat and tests whether its expression correlates with transpiration efficiency (as evidenced by stomatal density, stomatal conductance, carbon isotope discrimination) and yield. The goal is to establish whether the *TaER* genes could be used in approaches to improve transpiration efficiency and yield in wheat.

## Materials and Methods

### Plant material, growth conditions and sampling

Forty-eight bread wheat varieties with diverse carbon isotope discrimination (CID) values were sown in October, 2013 in the experimental field at Northwest A&F University, Yangling, Shaanxi, China (N 34°10’, E 108°10’, 526 m elevation). Details on the 48 varieties are provided in S1 Table; most of them are from the main wheat production areas of China, with two varieties (Drysdale and Quarrion) from Australia, which were characterized with low CID and high TE. Each variety was sown in 3 rows of 2 m length, with 25 cm between rows and 6.7 cm between plants. The wheat was grown without irrigation and dependent on the soil moisture and rainfall. The flag leaves of three plants of each variety were collected at heading (Z55) and grain-filling (Z73) stages [9], respectively. Epidermal samples taken from each leaf were used for monitoring the stomata density, and the remainder of each sample was quickly frozen in liquid nitrogen and stored at -80°C for preparation of total RNA.

### Sequences identification and analysis of *TaER* genes

Two *ER* sequences from *Triticum aestivum* (*ER2* from chromosome 7B, JQ599261.2 and *ER1* from chromosome 7D, JQ599260.2 [10]) were used to search for additional wheat *TaER* sequences with the BLASTN program on the URGI wheat genome database (http://urgi.versailles.inra.fr/Platform). Homologous sequences of *TaER* with at least 90% similarity were found on the chromosomes 6 and 7. These sequences were assembled with Geneious 6.0.5 software (Biomatters Ltd., USA), based on *ER1*, *ER2* and sequences from *T*. *urartu* and *Ae*. *tauschii* (progenitors of the A and D genomes, respectively) [11,12].

The phylogenetic tree of the six cDNA sequences was obtained using the Geneious Tree Builder software, and the amino acid sequences were deduced from the cDNA sequence of *TaER* genes. The functional domains of TaER were identified by a BLAST survey with the deduced proteins in SMART databank (http://smart.embl-heidelberg.de) and the Conserved Domain Search (http://www.ncbi.nlm.nih.gov/Structure/cdd/wrpsb.cgi).

### RNA extraction and cDNA synthesis

Total RNA was extracted according to the manufacturer’s recommendation using RNeasy Plant Mini Kit (QIAGEN, Germany). RNA concentration and purity were tested by Gene Quant Pro spectrophotometry (Amersham Biosciences, USA) and agarose gel electrophoresis. cDNA was synthesized with oligo(dT)_12–18_ by Super Script III Reverse Transcriptase (Invitrogen, USA) according to the manufacturer’s instructions. cDNA was stored at -20°C and then used for amplification.

### Expression analysis of *TaER* by qRT-PCR

The six cDNA sequences of the two *TaER* genes were aligned to design specific primers for *TaER1* or *TaER2*, selecting regions with high similarity among the three copies of each gene but with diversity between *TaER1* and *TaER2*. These primers were used to differentiate the specific expression of *TaER1* and *TaER2* in the flag leaves of the 48 wheat varieties at heading (Z55) and grain-filling (Z73) stages. Three housekeeping genes of *TaActin*, *TaSand* and *TaCell* were used to standardize the background expression in wheat. Details of the primers used for *TaER1*, *TaER2*, *TaActin*, *TaSand* and *TaCell* were listed in S2 Table. Each cDNA sample was used for three technical replicates according to the specifications of the SYBR Premix ExTaq Kit (TaKaRa, China) using the real time PCR system ABI 7300 (Applied Bio systems, USA). The reaction system included 10μl 2×SYBR MIX, 0.3μl for each of the forward and reverse primer, 2μl (50ng) template cDNA, ddH_2_O up to 20μl. The amplification procedure included an initial step of 95°C for 20s, followed by 40 cycles of 95°C for 5s, 61°C for 30s. Data was analyzed using the formula:

$$NE=\frac{{\left(E_{X}\right)}^{-Ct,X}}{{\left(E_{R}\right)}^{-Ct,R}}$$

Where *NE* is the relative expression of target gene, *E* is the primer efficiency, *Ct* value is collected where the fluorescence is above the threshold value, *X* indicates values from the target gene, *R* indicates the geometric mean of values from the three reference genes [13, 14, 15].

### Measurement of stomatal density and flag leaf area

Leaf epidermal samples of the 48 wheat varieties were collected at the grain-filling (Z73) stage, from both the adaxial (top) and abaxial (bottom) surface of flag leaves from three plants. The leaf surface was brushed with 1 cm^2^ of transparent nail polish for about 20 s, and covered with sellotape avoiding veins, if possible. The sellotape was then removed and placed on a microscope slide. Leaf epidermal samples were observed with a Zeiss Axiophot upright light microscope (Zeiss, Germany). Images were recorded using a QImaging Retiga Exi CCD digital camera (QImaging, Canada) and the MetaMorph Microscopy Automation & Image Analysis software (Molecular Devices, USA). An epidermal area free of debris was selected and oriented to allow as many stomata as possible inside the area of the image acquired (viewing area). Three images were collected from each slide. The total number of stomata in each image was counted and the average stomatal density (SD) of each flag leaf was estimated using the formula:

$$SD(No.mm^{-2})=\frac{Number\;of\;stomata\;(No.)}{View\;area\;(mm^{2})}$$

The flag leaves of five plants in each plot were measured to investigate flag leaf area (FLA) at the grain-filling stage (Z73) using the formula:

$$FLA\;(cm^{2})=leaf\;length\times leaf\;width\times 0.8$$

where leaf length and leaf width are the longest and widest dimensions of the flag leaf. [16]

### Measurement of stomatal related traits

At heading (Z55) and grain-filling (Z73) stages, the photosynthesis rate (A), stomatal conductance (gs), transpiration rate (E) and instant water use efficiency (WUEi) of the flag leaf were measured with a photosynthesis system (Li-6400, USA). The leaves were wide enough to completely fill the chamber area. Conditions in the leaf chamber were as reference CO_2_ concentration = 400 μmol mol^-1^, PPFD = 1800 μmol m^-2^ s^-1^, relative humidity 50–70% and block temperature = 20°C. Photosynthetic traits were measured for five plants of each of the 48 wheat varieties between 9:00 and 11:00 am in sunny and windless weather. After the measurements were concluded, samples were taken for *TaER* expression analyses.

### Measurement of CID, BYPP and GYPP

At the Z75 developmental stage, the flag leaves of three plants were collected and dried to constant weight, ground into fine powder and sent for measurement of δ^13^C using an Isotopic-Ratio Mass Spectrometer (Delta-V advantage, Germany) in the Lab of Stable Isotopes, Chinese Academy of Forestry Sciences (Beijing). An additional standard, Pee Dee Belemnite (PDB), was also measured. The CID value (Δ) was estimated using the formula:

$$\Delta \;(‰)=\frac{\left(\delta \text{a}-\delta \text{s}\right)}{\left(1+\delta \text{s}\right)}\times 1000$$

Where δs is the δ^13^C of the sample, and δa is the δ^13^C of atmospheric CO_2_, δa = -8 ‰ [17].

After harvest (Z93), biomass yield per plant (BYPP) and grain yield per plant (GYPP) were also investigated as described by Chen et al. [18], using five replicate plants for each of the 48 wheat varieties.

### Data analysis

Analysis of variance was used to assess variation of *TaER* expression, transpiration efficiency related traits, CID values and yield-related traits among the 48 wheat varieties. Varieties were grouped according to *TaER* expression following hierarchical cluster analysis of both *TaER1* and *TaER2* expression at the Z55 and Z73 developmental stages. Correlation analysis was performed between *TaER* expressions and these measured traits using the Pearson Product Moment Correlation test. All of the analyses used the SPSS Statistics Software version 19.0 (IBM SPSS Statistics, USA).

## Results

### Characterization of *TaER* genes in wheat

**Fig 1:**
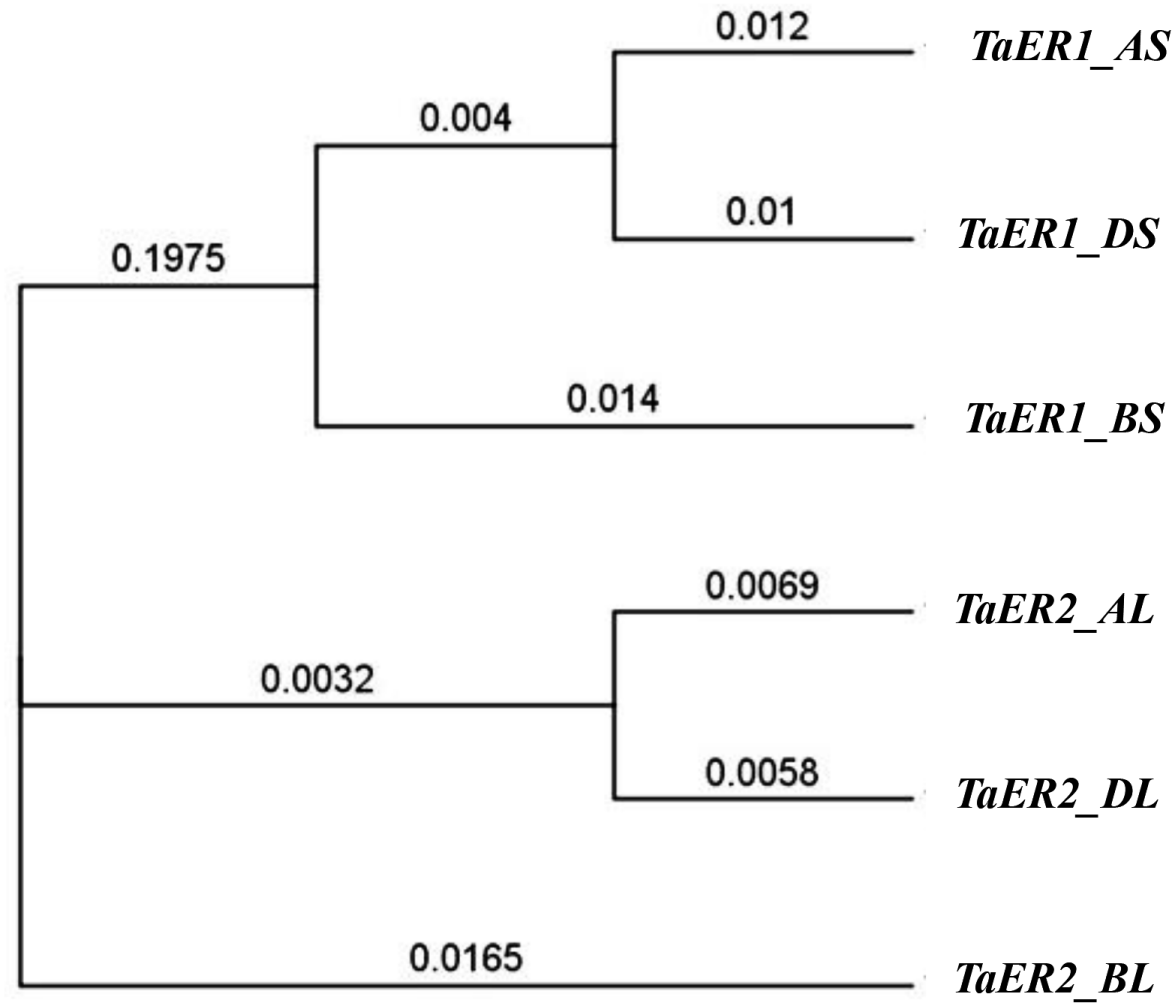
Phylogenetic tree of *TaER* genes family. Two genes (*TaER1* and *TaER2*) are encoded by each of the three genomes A, B and D (short arm of chromosome 7 for *TaER1* and long arm of chromosome 6 for *TaER2*). Six copies of cDNA sequences of *TaER* were aligned, and phylogenetic links were obtained based on their homology. Numbers relate to the phylogenetic distance between each of the *TaER* gene copies.

In previous work, the sequences of *ER2* (*TaER_B1*) and *ER1* (*TaER_D1*) were isolated by homology-based cloning with *ER* family genes, and localized on chromosomes 7B and 7D of bread wheat by using the nullisomic-tetrasomic lines of Chinese Spring [10]. A BLAST search using those cDNA sequences in the URGI databank revealed homologous sequences of *TaER* on the short arm of chromosome 7 and the long arm of chromosome 6. Copies of each gene, corresponding to the three bread wheat genomes (A, B and D,) were identified. *TaER1* was located on the short arm of chromosome 7 in all three genomes and the genes were named *TaER1_AS*, *TaER1_BS* (equivalent to *TaER_B1*) and *TaER1_DS* (equivalent to *TaER_D1*). *TaER2* was located on the long arm of chromosome 6 in all three genomes and the genes were named *TaER2_AL*, *TaER2_BL* and *TaER2_DL*. Alignment between *TaER1* and *TaER2* showed 75.4% identical nucleotides, while the three copies of *TaER1* and *TaER2* showed 94.4% and 95.9% identical nucleotides, respectively. Both *TaER1* and *TaER2* contained 27 exons and 26 introns, cluster analysis indicated that the copies of *TaER* genes were divided into two groups (corresponding to each of *TaER1* and *TaER2*), and in each case the gene copies encoded by genome A were more similar to those encoded by genome D than to those encoded by genome B (Fig 1).

**Fig 2:**
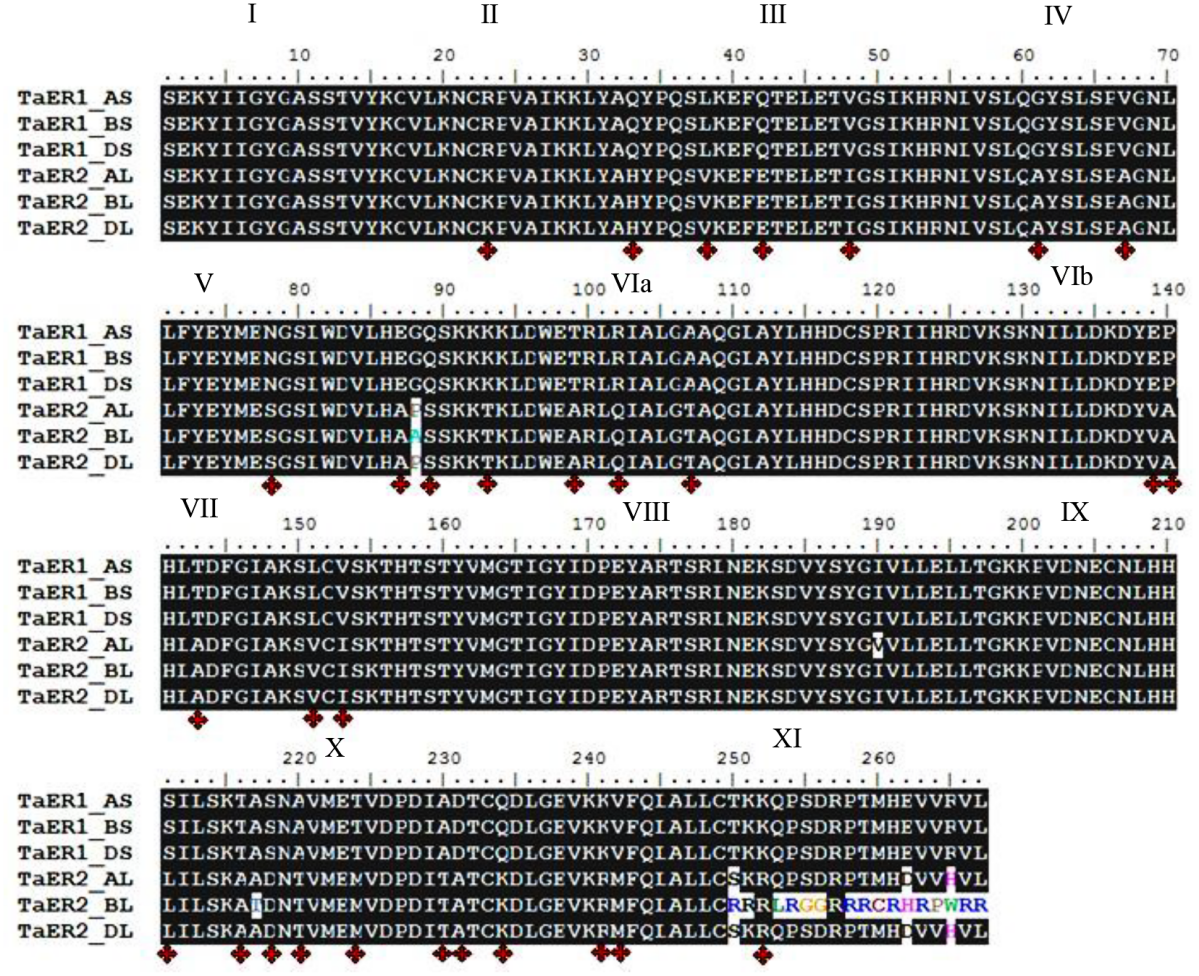
Sequence alignment of *TaER* Ser/Thr kinase domain. Red stars indicate different amino acid residues between chromosomes 6 and 7. The lack of black background indicates different amino acids among the genomes A, B and D. Roman numerals on top of sequences indicate the 11 subdomains of TaER protein kinases.

Alignment of the deduced amino acid sequences of TaER revealed a predicted extracellular domain consisting of leucine-rich repeat (LRR) elements, a Serine/Threonine (Ser/Thr) protein kinase domain at the C-terminal, and a transmembrane region from amino acids 579 to 589, which shared the same conserved domains as other ER family proteins. Alignment of the predicted sequences of TaER copies showed the high homology in the kinase region but the variable extracellular and transmembrane regions. In TaER1, there were 93% identical amino acids across genomes with identical amino acids in the kinase region; in TaER2, there were 93.2% identical amino acids across genomes with 18 amino acids varied in the kinase region. The Ser/Thr kinase region showed 268 highly conserved residues and the 11 typical sub-domains were recognized by homology to other ER family proteins (Fig 2).

### Expression of *TaER1* and *TaER2* in flag leaves of 48 wheat varieties

**Fig 3:**
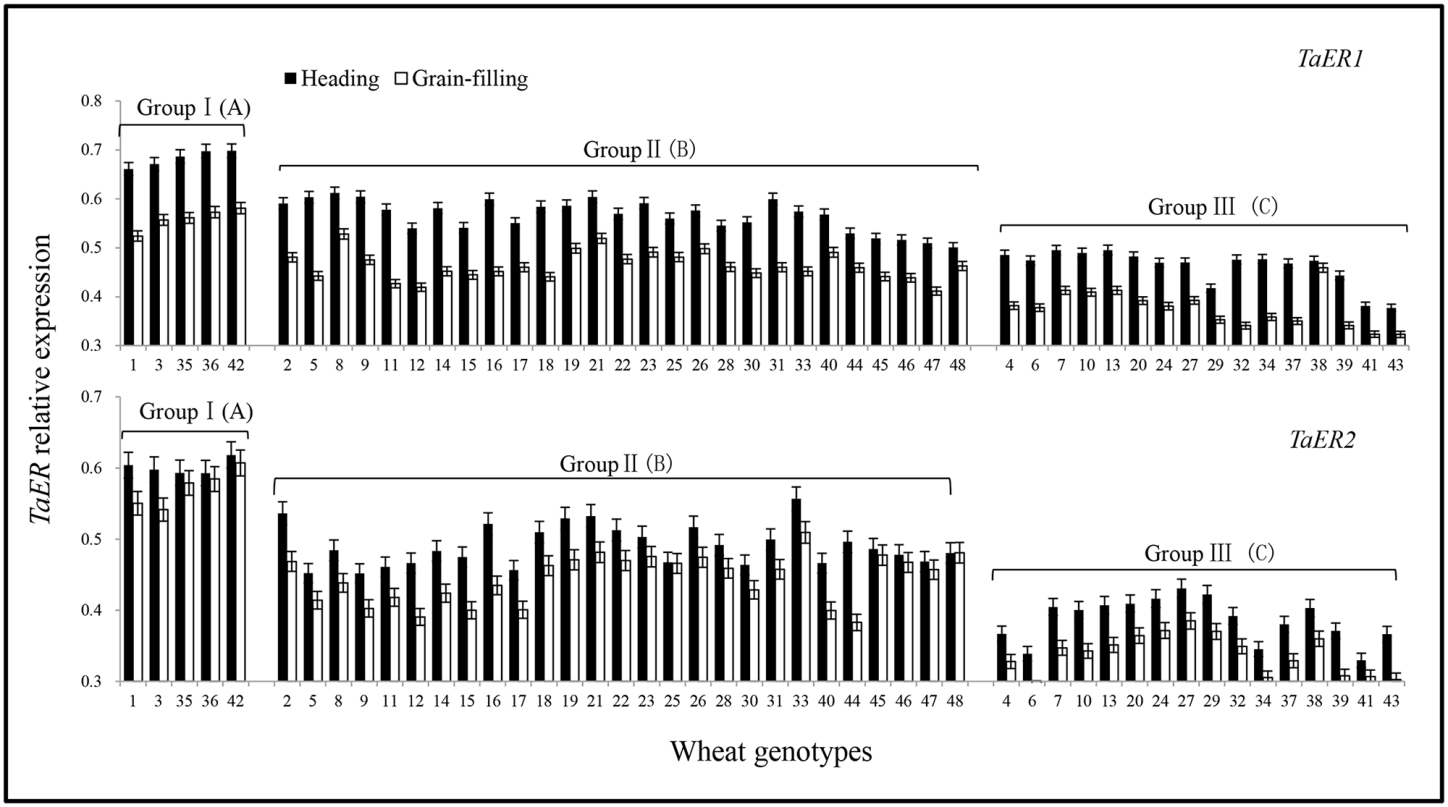
Relative expression of *TaER1* and *TaER2* in flag leaves of 48 wheat genotypes at heading (Z55) and grain-filling (Z73) stage. Group I: high *TaER* expression; Group II: intermediate *TaER* expression; Group III: low *TaER* expression. Uppercase letters represent significant differences among the three groups (*P*<0.01). The reference genes were *TaActin*, *TaCell* and *TaSand*. Values are presented with the mean ± SD calculated from the formula [13, 14, 15]:
$$NE=\frac{{\left(E_{X}\right)}^{-Ct,X}}{{\left(E_{R}\right)}^{-Ct,R}}$$

**Fig 4:**
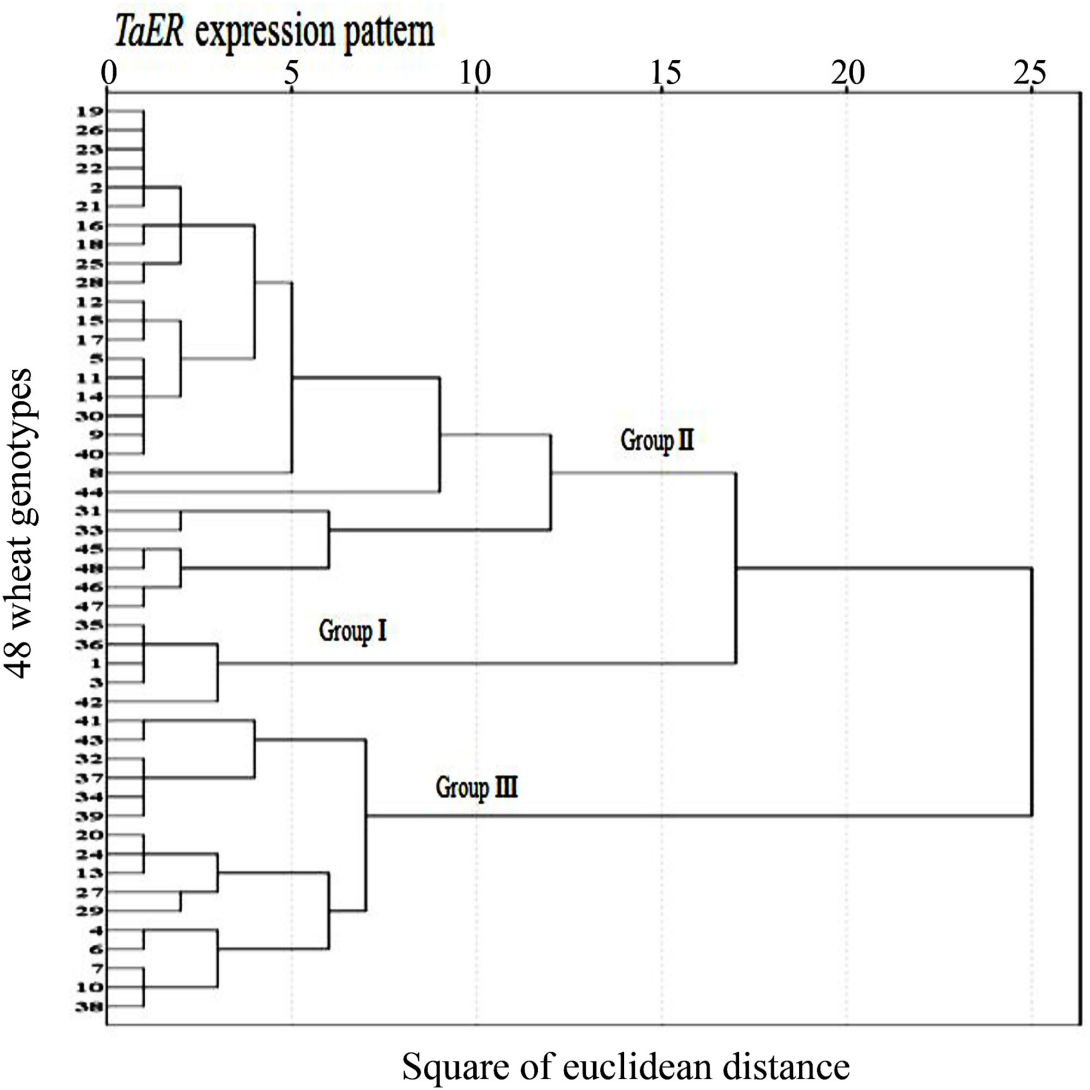
Cluster analysis of *TaER* expression in the 48 wheat genotypes (*P*<0.01). Group I: high *TaER* expression; Group II: intermediate *TaER* expression; Group III: low *TaER* expression. The numbers of Y axis present the code of the diverse wheat varieties (S1 Table), X axis presents the square of euclidean distance.

The expression levels of *TaER1* and *TaER2* in wheat flag leaves were higher at heading (Z55) stage than at grain-filling (Z73) stage, with similar expression patterns in both *TaER1* and *TaER2* at the two stages (Fig 3, S3 Table). Significant variations were observed on the expression levels of *TaER1* and *TaER2* in the flag leaves at both stages among the 48 diverse bread wheat varieties (Fig 3, S3 Table). Cluster analysis on the expression levels of two *TaER* genes, classified these varieties into three groups as: 5 varieties with high *TaER* expression in group I, 27 varieties with intermediate *TaER* expression in group II and 16 varieties with low *TaER* expression in group III (Fig 4, S3 Table). The distribution of genotypes per group implied that there were relatively few genotypes with higher *TaER* expressions, but most genotypes with intermediate *TaER* expression across these wheat varieties.

At both developmental stages, the expression levels of *TaER1* and *TaER2* were significantly different (*P*< 0.01) among the three groups (Fig 3, Table 1). The expression of both genes was about 30–40% lower in group III compared to group I at both developmental stages. In each group, significant differences (P< 0.05) were also detected among different wheat varieties (S3 Table). The wheat variety Drysdale (No.42) showed the highest expression level of both *TaER* genes.

**Table 1:** *TaER* relative expression in the three groups of 48 wheat varieties

TaER genes	Development stage	Items	Grouping of 48 wheat varieties		
			Group I	Group II	Group III
*TaER1*	Heading(Z55)	Mean	0.68±0.004 A	0.57±0.004 B	0.46±0.005 C
	Heading(Z55)	Minimum	0.66	0.50	0.38
	Heading(Z55)	Maximum	0.70	0.61	0.50
	Grain-filling(Z73)	Mean	0.56±0.005 A	0.46±0.004 B	0.38±0.005 C
	Grain-filling(Z73)	Minimum	0.52	0.40	0.32
	Grain-filling(Z73)	Maximum	0.58	0.53	0.46
*TaER2*	Heading(Z55)	Mean	0.60±0.002 A	0.49 ±0.003 B	0.39±0.004 C
	Heading(Z55)	Minimum	0.59	0.44	0.33
	Heading(Z55)	Maximum	0.62	0.56	0.44
	Grain-filling(Z73)	Mean	0.57±0.006 A	0.44±0.003 B	0.34±0.004 C
	Grain-filling(Z73)	Minimum	0.55	0.38	0.29
	Grain-filling(Z73)	Maximum	0.61	0.51	0.39

Group I: high *TaER* expression; Group II: intermediate *TaER* expression; Group III: low *TaER* expression. Uppercase letters represent significant differences among the three groups (*P*<0.01).

### Correlation between *TaER* expression and TE related traits

**Fig 5:**
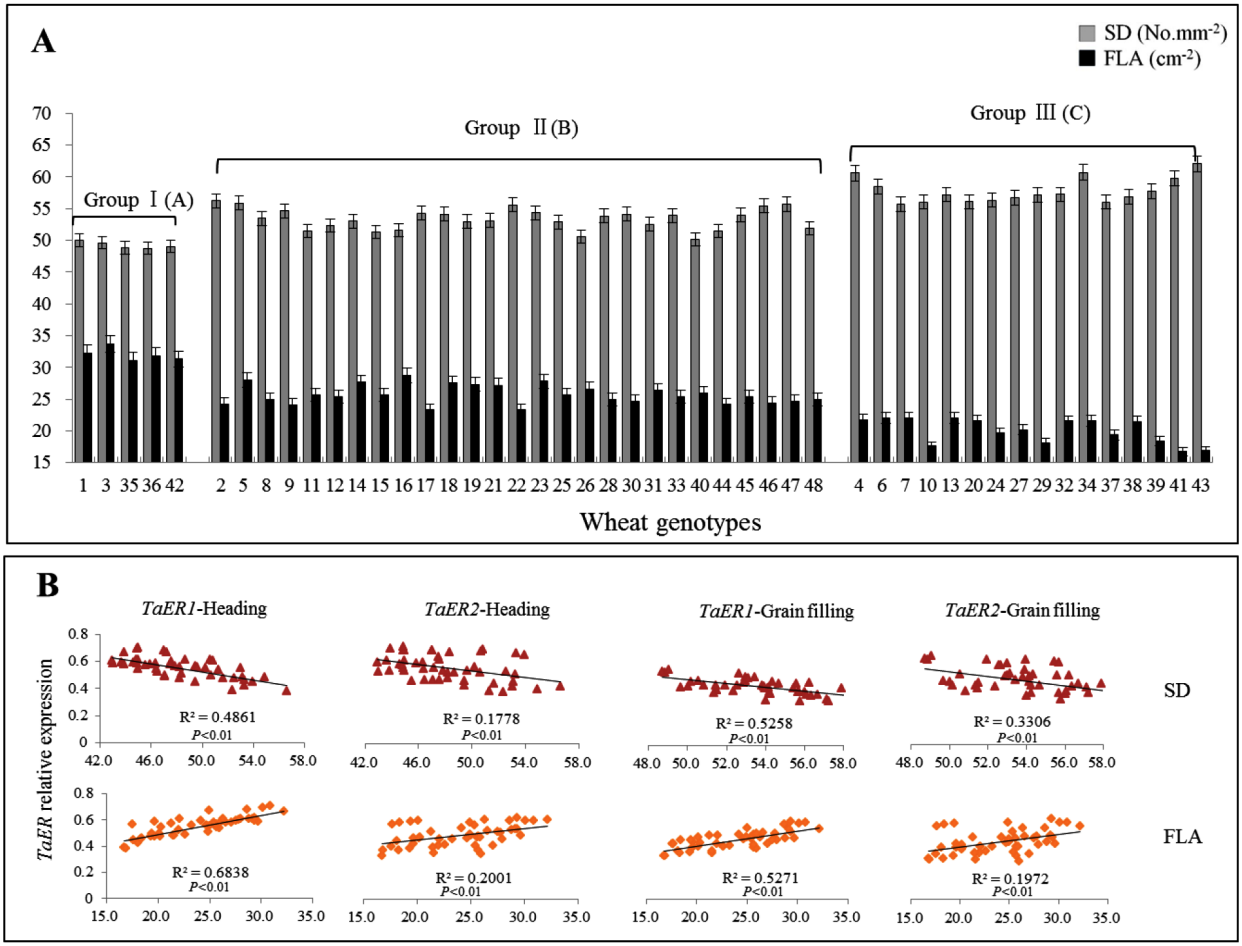
Correlation between *TaER* expression with SD of flag leaf and FLA. A: Stomatal density (SD) of flag leaf and flag leaf area (FLA) of the 48 wheat genotypes; B: Regression analysis between *TaER* expression with SD of flag leaf and FLA. Group I: high *TaER* expression; Group II: intermediate *TaER* expression; Group III: low *TaER* expression. Uppercase letters represent significant differences among the three groups (*P*< 0.01).

The stomatal density of flag leaf (SD) and flag leaf area (FLA) were significantly different among the 48 wheat varieties at the grain-filling stage (Z73) (S5 Table). SD and FLA were also significantly different (*P*< 0.01) among the three groups (Fig 5A, Table 2). SD was the lowest and FLA was the highest in the group of varieties with high *TaER* expression (group I), within this group, the varieties Fengchan3 (No. 36) and Shijiazhuang8 (No. 3) had the lowest SD and the highest FLA. At both heading (Z55) and grain-filling (Z73) stages, negative linear correlations were found between *TaER* expression and SD; whereas positive linear correlations were detected between *TaER* expression and FLA (Fig 5B, Table 3).

**Table 2:** Transpiration efficiency related traits and yield in the three groups of 48 wheat varieties

Traits	Grouping of 48 wheat varieties		
	Group I	Group II	Group III
SD (No.mm^-2^)	49.24±0.15 A	53.37±0.18 B	57.79±0.28 C
FLA (cm^2^)	32.02±0.24 A	25.69±0.16 B	20.03±0.28 C
A (μmol.m^-2^ s^-1^)	21.54±0.13 Aa	18.52±0.11 BCb	16.58±0.09 Cc
E (mmol.m^-2^ s^-1^)	2.76±0.03 Aa	3.70±0.04 BCb	4.19±0.05 Cc
WUEi (μmol.mmol^-1^)	6.79±0.07 Aa	5.67±0.04 BCb	4.95±0.05 Cc
CID (‰)	21.07±0.09 A	21.53±0.03 B	22.17±0.03 C
BYPP (g)	42.46±0.37 A	33.76±0.38 B	24.10±0.42 C
GYPP (g)	19.39±0.40 Aa	14.19±0.24 BCb	12.68±0.36 Cc
HI	0.46±0.04	0.43±0.01	0.54±0.02

Group I: high *TaER* expression; Group II: intermediate *TaER* expression; Group III: low *TaER* expression. Uppercase and lowercase letters represent significant differences among the three groups (uppercase *P*< 0.01; lowercase *P*< 0.05). SD: stomatal density (No. mm^-2^); FLA: flag leaf area (cm^2^); A: photosynthetic rate (μmol. m^-2^ s^-1^); E: transpiration rate (mmol. m^-2^ s^-1^); WUEi: instant water use efficiency (μmol mmol^-1^); CID: carbon isotopic discrimination (‰); BYPP: biomass yield per plant (g); GYPP: grain yield per plant (g); HI: harvest index.

**Table 3:** Correlation coefficient between *TaER* expressions with transpiration efficiency related traits and yield.

Trait	*TaER1*		*TaER2*	
	Heading(Z55)	Grain-filling(Z73)	Heading(Z55)	Grain-filling(Z73)
SD	-0.697**	-0.725**	-0.422**	-0.575**
FLA	0.727**	0.826**	0.447**	0.448**
A	0.216*	0.366*	0.129*	0.275*
gs	-0.059	0.069	-0.157	0.096
E	-0.376*	-0.486*	-0.210*	-0.304*
WUEi	0.227*	0.258*	0.142*	0.192*
CID	-0.752**	-0.793**	-0.524**	-0.578**
GYPP	0.573**	0.656**	0.319*	0.328*
BYPP	0.651**	0.658**	0.489**	0.494**
HI	0.202	-0.095	-0.246	-0.335*

Asterisks represent significant differences.

* *P*< 0.05;

** *P*< 0.01. SD: stomatal density (No. mm^-2^); FLA: flag leaf area (cm^2^); A: photosynthetic rate (μmol. m^-2^ s^-1^); gs: stomatal conductance (mmol. m^-2^ s^-1^); E: transpiration rate (mmol. m^-2^ s^-1^); WUEi: instant water use efficiency (μmol. mmol^-1^); CID: carbon isotopic discrimination (‰); BYPP: biomass yield per plant (g); GYPP: grain yield per plant (g); HI: harvest index.

**Fig 6:**
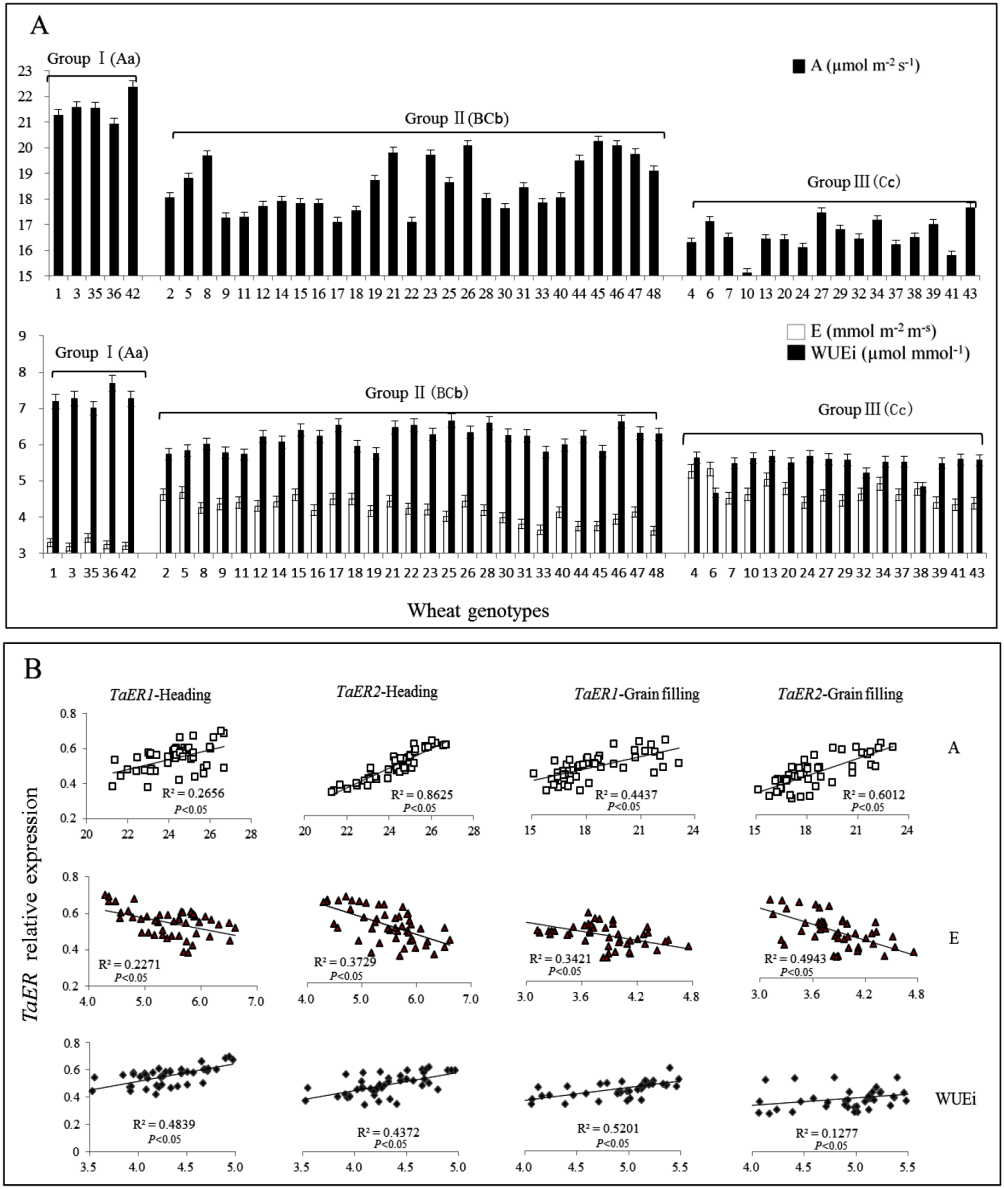
Correlation between *TaER* expression and transpiration efficiency related traits. A: Photosynthetic rate (A), transpiration rate (E) and instant water use efficiency (WUEi) of the 48 wheat genotypes; B: Regression analysis between *TaER* expression with A, E and WUEi. Group I: high *TaER* expression; Group II: intermediate *TaER* expression; Group III: low *TaER* expression. Upper and lowercase letters represent significant differences among the three groups (uppercase *P*< 0.01, lowercase *P*< 0.05).

Variation in photosynthesis rate (A), stomatal conductance (gs), transpiration rate (E) and instant water use efficiency (WUEi) among the 48 wheat varieties (S5 Table) at grain-filling stage (Z73) was closely correlated with *TaER* expression levels in the corresponding groups (Fig 6A, Table 2). Photosynthesis rate was higher, transpiration rate was lower and WUEi was higher in wheat varieties with high expression of *TaER*. The varieties Drysdale (No. 42) and Fengchan3 (No. 36) displayed the highest A and WUEi, and the variety Shijiazhuang8 (No. 3) had the lowest E.

Regression analyses confirmed that *TaER* expression was significantly and positively correlated with A and WUEi, while significantly and negatively correlated with E (Fig 6B, Table 3), but was not significantly correlated with gs at heading (Z55) and grain-filling (Z73) stages. The significance of these correlations was stronger for *TaER1* than for *TaER2*.

### Correlation between *TaER* expression and CID, BYPP or GYPP

**Fig 7:**
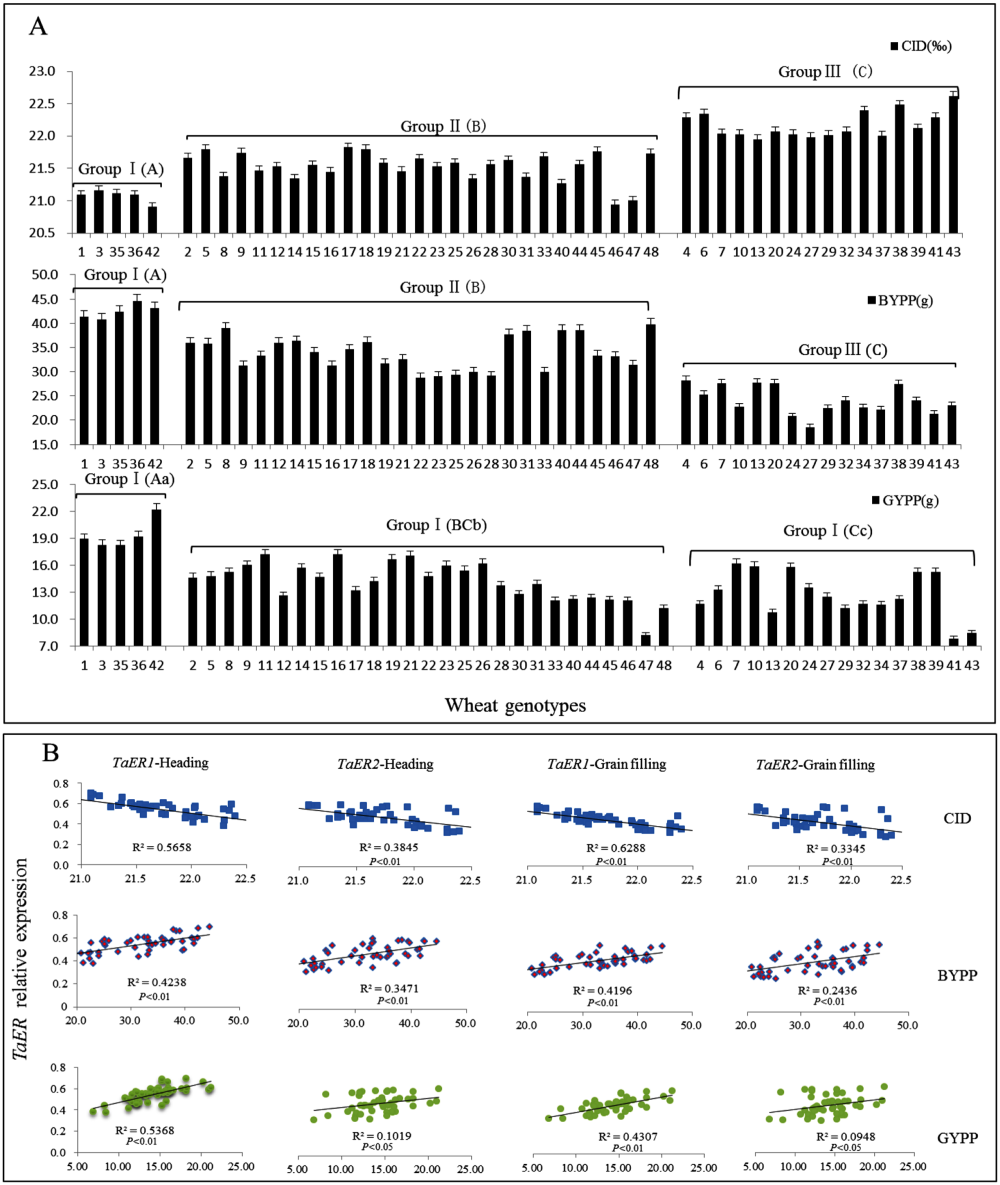
Correlation between *TaER* expression with CID, BYPP and GYPP. A: Flag leaf carbon isotope discrimination (CID), biomass yield per plant (BYPP) and grain yield per plant (GYPP) of the 48 wheat genotypes; B: Regression analysis between *TaER* expression with CID, BYPP and GYPP. Group I: high *TaER* expression; Group II: intermediate *TaER* expression; Group III: low *TaER* expression. Upper and lowercase letters represent significant differences among the three groups (uppercase *P*< 0.01, lowercase *P*< 0.05).

The carbon isotope discrimination (CID) of flag leaf was determined at grain filling (Z73) stage, and the biomass yield plant^-1^ (BYPP) and grain yield plant^-1^ (GYPP) were determined after harvest (Z93) (S6 Table). CID, BYPP and GYPP were significantly different (P< 0.05) between groups of wheat genotypes characterized by different levels of *TaER* expression (Fig 7A, Table 2). In general, cultivars with high *TaER* expression had low CID and high BYPP and GYPP, with group I varieties Drysdale (No. 42) and Fengchan3 (No. 36) showing the lowest CID and the highest BYPP. Correlation analysis indicated that CID was negatively correlated with *TaER* expression, but BYPP and GYPP were positively correlated (Fig 7B, Table 3). At grain-filling (Z73) stage, *TaER1* expression was more strongly correlated with CID, BYPP and GYPP than *TaER2* (Table 3).

### Correlation analysis among TE- and yield- related traits

Correlation analysis between the measured traits was performed in the 48 wheat varieties (Table 4). The results showed that there were significant correlations (P< 0.05) among most traits, but gs did not correlate with SD, FLA or CID. Similarly, not all traits correlated with harvest index (HI), but significant correlations with HI were observed for A, gs and GYPP. GYPP was positively correlated with FLA, A, gs, WUEi, BYPP and HI, but negatively correlated with SD, E and CID.

**Table 4:** Correlation coefficient between transpiration efficiency related traits and yield.

	FLA	SD	A	gs	E	WUEi	CID	BYPP	GYPP	HI
**FLA**		-0.543**	0.467**	-0.154	-0.625**	0.637**	-0.684*	0.624**	0.604**	0.002
**SD**	-0.543**		-0.578*	-0.128	0.782**	-0.650**	0.775*	-0.639**	-0.539**	-0.028
**A**	0.467**	-0.578*		0.217*	-0.705**	0.618**	-0.600**	0.556**	0.348*	0.369*
**gs**	-0.154	-0.128	0.217*		0.306*	-0.260*	0.038	0.316*	0.246*	0.325*
**E**	-0.625**	0.782**	-0.505*	0.306*		-0.688**	0.760**	-0.557**	-0.602**	0.139
**WUEi**	0.637**	-0.650**	0.618**	-0.260*	-0.688**		-0.699**	0.481**	0.620**	0.003
**CID**	-0.684*	0.775*	-0.600**	0.038	0.760**	-0.699**		-0.600*	-0.608*	0.201*
**BYPP**	0.624**	-0.639**	0.556**	0.316*	-0.557**	0.481**	-0.600*		0.324*	-0.107
**GYPP**	0.604**	-0.539**	0.348*	0.246*	-0.602**	0.620**	-0.608*	0.324*		0.375**
**HI**	0.002	-0.028	0.369*	0.325*	0.139	0.003	0.201	-0.107	0.375**	

Asterisks represent significant differences.

* *P*< 0.05;

** *P*< 0.01. SD: stomatal density (No. mm^-2^); FLA: flag leaf area (cm^2^); A: photosynthetic rate (μmol. m^-2^ s^-1^); gs: stomatal conductance (mmol. m^-2^ s^-1^); E: transpiration rate (mmol. m^-2^ s^-1^); WUEi: instant water use efficiency (μmol. mmol^-1^); CID: carbon isotopic discrimination (‰); BYPP: biomass yield per plant (g); GYPP: grain yield per plant (g); HI: harvest index.

## Discussion

ER has multiple effects on plant development, growth and physiology. Gene Ontology analysis revealed several pathways potentially mediated by ER [1]. Although most functional analyses have been performed in *Arabidopsis*, the presence of ER and ER-Like members in several crop species suggests that it may provide a novel breeding target. This study provided a detailed characterization of *TaER* genes in bread wheat and showed that a correlation exists between *TaER* expression and transpiration efficiency traits related to development and productivity of wheat.

### *TaER* genes in wheat

Six copies of *TaER*, homologues of the well-characterized *ERECTA* (*ER*) genes in *Arabidopsis*, were identified in the genome of bread wheat (using data currently available in the public domain). The *TaER1* and *TaER2* genes were located on chromosomes (6 and 7) and occurred in the three wheat genomes (A, B and D). The six copies had similar predicted amino acid sequences, reflecting the relatively conserved evolutionary history of the ER family in bread wheat [2]. Cluster analysis suggested that sequences encoded by genomes A and D are more closely related than genome B. These results would suggest that the three copies maybe not be complementary, but likely have parallel functions. This hypothesis could be tested in future experiments by silencing the individual *TaER* genes.

### *TaER* expression in wheat

Variations were observed in the expression levels of *TaER1* and *TaER2* among the 48 wheat genotypes. *TaER1* and *TaER2* had conserved amino acid sequences and probably overlapping functions. The 48 wheat varieties were clustered into three groups as high, moderate and low of *TaER* expression levels; these significant variations may regulate the change of agronomical traits during the wheat development. Most studies on the expression of *ERECTA* have been carried out in mutants of *Arabidopsis* and these did not provide evidence on the expressional diversity of this gene in a panel of germplasm. In this study, the intraspecific diversity in the expression of *TaER* genes has been shown in flag leaves of 48 wheat genotypes at two growth stages, which are sufficient to provide a potentially useful estimate of intra-specific phenotypic variability. This finding is in agreement with results for anatomical and physiological changes following overexpression of *PdERECTA* in *Arabidopsis* [7]. Moreover, as the total expression of the three copies in A, B and D genome of *TaER1* and *TaER2* were evaluated in this study, to clarify whether there were variations among the three copies, the expression patterns of the three copies in A, B and D genome for *TaER1* and *TaER2* were further evaluated in four genotypes with two for high expression and two for low expression of *TaER1* and *TaER2*, which showed highly similar expression patterns with that of *TaER1* and *TaER2* common expression, although there were variations among the three copies, especially for that of *TaER1* at heading stage (S4 Table), this suggested that the total expression of *TaER1* and *TaER2* could be used to evaluate the diversity of *TaER* expression in different wheat varieties, and the variation seen within the 48 genotypes may be due to *TaER* expression diversity and that *TaER* is a critical factor in the wheat leaf development.

### Effect of TaER on leaf anatomy

Compelling evidence already suggests that ER affects leaf development and stomata formation [19]. Here, SD and FLA varied greatly among the high, medium and low *TaER* expression groups within the 48 wheat genotypes, the expression of both *TaER1* and *TaER2* were negatively and positively correlated with SD and FLA, respectively. Since leaf anatomy is a primary determinant of water relations between the plant and the environment [20] and TaER expression is significantly correlated with leaf anatomy of SD and FLA traits, so we predict TaER similarly regulates the development of epidermal cells and affects stomatal density in wheat leaves. This could be investigated further in transgenic wheat plants in which TaER is either overexpressed or silenced.

Correlation of *TaER* expression with flag leaf traits was stronger at grain-filling (Z73) stage than that at heading (Z55) stage. It is possible that the development of leaf anatomy at heading stage is regulated by a combination of TaER in addition to other factors, such as TMM (TOO MANY MOUTHS) and EPF (EPIDERMAL PATTERNING FACTOR) [20]. The stronger correlation coefficients found for *TaER1* than *TaER2* indicates that the former has a stronger effect on the development of wheat leaf anatomy.

### Association of *TaER* expression with transpiration efficiency related traits

The variation in SD and FLA probably led to the diversity in transpiration efficiency related traits seen among the 48 genotypes. In this study, *TaER* expression was positively correlated with A and WUEi, but negatively correlated with E. This is in agreement with previous studies in *Arabidopsis*. The link between *TaER* expression and photosynthesis rate offers another strategic target for breeding or biotechnological approaches to increase photosynthesis in wheat. However, there was only a weak correlation between *TaER* expression and gs. A possible reason might be that gs is not only determined by genetic variation but is also strongly influenced by environmental factors [21]. These results are consistent with *TaER* expression indirectly controlling photosynthesis.

As with SD and FLA, the correlation coefficients between *TaER* expression and transpiration efficiency related traits were higher at grain-filling (Z73) stage than that at heading (Z55) stage. At heading stage, the wheat organs were under rapid change, hence measured data were more variable. At grain-filling (Z73) stage, almost all of the vegetative organs were formed, measured data were less variable and correlation coefficients were higher. Moreover, *TaER1* expression was more strongly correlated with the diverse transpiration efficiency related traits than *TaER2*. These results support that *TaER1* has a stronger effect on the regulation of wheat development than *TaER2*. Silencing studies within the TaER family would provide more evidence for the functional effects of *TaER1* and *TaER2*.

### Regulation of transpiration efficiency related traits and yield by TaER

CID provides a reliable integrated measurement of A, E and WUE, and is proposed as an indicator of transpiration efficiency in the long term, avoiding issues associated with instant measurements made under varying environmental conditions [21]. *AtER* was suggested as the major gene contributing 20–60% of CID variation in *Arabidopsis*. Masle et al. [1] showed that the gene product of *ER* influences significantly CID and WUE in *Arabidopsis*, with lower transcript levels relating to higher CID and lower WUE. Here, it was also found that genotypes with higher *TaER* expression levels had lower CID and higher WUEi of flag leaf in bread wheat. Correlation analyses also suggested that *TaER* expression was positively correlated with BYPP and GYPP. It is proposed that *TaER* expression could be used as a rapid and reliable criterion for selecting superior genotypes for wheat breeding.

## Conclusions

Anatomical and physiological traits determining transpiration efficiency, such as stomatal density, photosynthetic and transpiration rates, carbon isotope discrimination and yield traits were measured to evaluate the correlation between *TaER* expression with transpiration efficiency related traits and yield in 48 wheat varieties. *TaER* expression was associated with higher flag leaf area (FLA), photosynthetic rate (A) and WUEi, lower stomatal density (SD), transpiration rate (E) and CID, higher biomass yield per plant (BYPP) and grain yield per plant (GYPP). In addition, *TaER1* had a stronger effect at grain-filling (Z73) stage on the development of bread wheat than *TaER2*. The functions of the two genes could be further investigated by independent silencing of the *TaER* genes.

## Supporting Information

S1 Table Name and planting region of the 48 wheat varieties. (PDF)

S2 Table Primer sequences used for expression analysis of *TaER1* and *TaER2*. (PDF)

S3 Table TaER relative expression in 48 wheat varieties. (PDF)

S4 Table Relative expression of three homologous copies in the A, B and D genome of *TaER1* and *TaER2* in 4 wheat varieties. (PDF)

S5 Table Transpiration efficiency related traits of 48 wheat varieties at grain- filling (Z73) stage. (PDF)

S6 Table Carbon isotope discrimination and yield-related traits of the 48 wheat varieties. (PDF)
