# Supplementary material for: TaER Expression Is Associated with Transpiration Efficiency Traits and Yield in Bread Wheat
Source: PLoS One. 2015 Jun 5;10(6):e0128415. doi: 10.1371/journal.pone.0128415 (PMC4457575; doi:10.1371/journal.pone.0128415)
Supplement: S1 Table — (PDF) [file pone.0128415.s001.pdf]

S1 Table. Name and planting region of the 48 wheat varieties

No.	Genotype	Planting Region*	Province of Origin
1	Yunhan 22-33	Huang-huai WWR	Shanxi
2	Luohan 6	Huang-huai WWR	Henan
3	Shijiazhuang 8	Huang-huai WWR	Hebei
4	Hanyou 98	Huang-huai WWR	Shandong
5	Zhonghan 110	Northern WWR	Beijing
6	Youmai 2	Northern WWR	Shandong
7	Changwu 134	Huang-huai WWR	Shaanxi
8	Jinmai 47	Northern WWR	Shanxi
9	Changwu 863	Huang-huai WWR	Shaanxi
10	Changwu 521-7	Huang-huai WWR	Shaanxi
11	Shan 229	Huang-huai WWR	Shaanxi
12	Xiaoyan 6	Huang-huai WWR	Shaanxi
13	Shanmai 168	Huang-huai WWR	Shaanxi
14	Pubing 201	Huang-huai WWR	Shaanxi
15	Shan 512	Huang-huai WWR	Shaanxi
16	Xiaoyan 22-3	Huang-huai WWR	Shaanxi
17	Pubing 143	Huang-huai WWR	Shaanxi
18	Xinong 389	Huang-huai WWR	Shaanxi
19	Liken 2	Huang-huai WWR	Shaanxi
20	Lantian 10	Huang-huai WWR	Shaanxi
21	Xinong 811	Huang-huai WWR	Shaanxi
22	Jing 411	Northern WWR	Beijing
23	Jinan 18	Northern WWR	Shandong
24	Qinnong 712	Huang-huai WWR	Shaanxi
25	Jiufeng 22	Huang-huai WWR	Shaanxi
26	Ligao 6	Huang-huai WWR	Shaanxi
27	Changwu 58-61	Huang-huai WWR	Shaanxi
28	Jing 2001	Northwest WWR	Gansu
29	Mianyang 11	Southwestern WWR	Sichuan
30	Jining 13	Northern WWR	Shandong
31	Xinmai 19	Huang-huai WWR	Henan
32	Zhou 17	Huang-huai WWR	Henan
33	Zhou 19	Huang-huai WWR	Henan
34	Yuanfeng 175	Huang-huai WWR	Shaanxi
35	Yuanfeng 139	Huang-huai WWR	Shaanxi
36	Fengchan 3	Huang-huai WWR	Shaanxi
37	Xinong 889	Huang-huai WWR	Shaanxi
38	Xinong 979	Huang-huai WWR	Shaanxi
39	Xinong 928	Huang-huai WWR	Shaanxi
40	Xiaoyan 81	Northern WWR	Beijing
41	Shan 7859	Huang-huai WWR	Shaanxi
42	Drysdale	Australia SWR	New south wales

43	Quarrion	Australia SWR	New south wales
44	Luomai 21	Huang-huai WWR	Henan
45	Jinmai 33	Northern WWR	Shanxi
46	Xifeng 20	Huang-huai WWR	Gansu
47	Ningchun 45	Northwest SWR	Ningxia
48	Hongmangmai	Northwest SWR	Ningxia

*WWR: Winter wheat region; SWR: Spring wheat region.
