# Supplementary material for: TaER Expression Is Associated with Transpiration Efficiency Traits and Yield in Bread Wheat
Source: PLoS One. 2015 Jun 5;10(6):e0128415. doi: 10.1371/journal.pone.0128415 (PMC4457575; doi:10.1371/journal.pone.0128415)
Supplement: S2 Table — (PDF) [file pone.0128415.s002.pdf]

S2 Table. Primer sequences used for expression analysis of *TaER1* and *TaER2*

Primer	Primer sequence (5'−3')	Usage
TaER1-F	AACTGAGCTTGAGACTGTCGGC	qRT-PCR of *TaER1* (chromosome 7)
TaER1-R	CCAGAGGCTGCCATTTTCCATG	
TaER2-F	GAACTTGAGGGAACGATACCTG	qRT-PCR of *TaER2* (chromosome 6)
TaER2-R	AAGCTTGTGCAATGAACGTGGG	
TaER1_AS-F	CGGGCTCAACCTCGAGGGCGAAATCTCT	qRT-PCR of *TaER1* in relation to the three orthologs in A, B and D genome
TaER1_AS-R	GCGTCTTAATCGATGAGCAATCCCCAATCTCG	
TaER1_BS-F	CTCCCACAACACAGAGTAGGACTTTGAGTCT	
TaER1_BS-R	CTACTCGTCTGACTGACTACCTGCTTGC	
TaER1_DS-F	ACAACACGGAGTAGGACGGAGAGGCC	
TaER1_DS-R	TTGCTACTACTCGTCTGACCGACTACCTGG	
TaER2_AL-F	GGCTACATTGACCCTGAGTATGCGCGG	qRT-PCR of *TaER2* in relation to the three orthologs in A, B and D genome
TaER2_AL-R	TTGTCAACTGGCTTCTTCCCAGTCAGCAG	
TaER2_BL-F	TTGCTGACTGGGAAGAAGCCAGTTGACAAC	
TaER2_BL-R	GGCACCGTCGCCTTCCTCCTCTTAGCCG	
TaER2_DL-F	TCCCCGGGCTACTGCTCCTGGCT	
TaER2_DL-R	GGAGGACAGGTTCAACATTTTAAGCGACC	
TaActin-F	TTGCTGACCGTATGAGCAAG	Reference genes in wheat
TaActin-R	ACCCTCCAATCCAGACACTG	
TaSand-F	TGCCTTGCCCATAAGAAATC	
TaSand-R	GTGCGGACCAGTTGCTTTAT	
TaCell-F	GAGGAGGATGAGGTGGATGA	
TaCell-R	CCTGGTACTTGCGGATGTCT	
