# Supplementary material for: TaER Expression Is Associated with Transpiration Efficiency Traits and Yield in Bread Wheat
Source: PLoS One. 2015 Jun 5;10(6):e0128415. doi: 10.1371/journal.pone.0128415 (PMC4457575; doi:10.1371/journal.pone.0128415)
Supplement: S3 Table — (PDF) [file pone.0128415.s003.pdf]

S3 Table. *TaER* relative expression in 48 wheat varieties

Group	Genotype No.	*TaER1*		*TaER2*	
		Heading(Z55)	Grain-filling(Z73)	Heading(Z55)	Grain-filling(Z73)
I	1	0.6611 d	0.5242 e	0.6041 b	0.5505 c
	3	0.6715 c	0.5571 c	0.5979 b	0.5417 d
	35	0.6868 b	0.5613 c	0.5933 b	0.5791 b
	36	0.6977 a	0.5730 b	0.5929 b	0.5845 b
	42	0.6987 a	0.5812 a	0.6183 a	0.6072 a
II	2	0.5906 g	0.4808 h	0.5364 d	0.4687 g
	5	0.6035 f	0.4427 k	0.4522 o	0.4141 m
	8	0.6121 e	0.5283 d	0.4845ij	0.4385 i
	9	0.6046 f	0.4753 h	0.4519 o	0.4026 n
	11	0.5780 h	0.4268 l	0.4610mno	0.4181 l
	12	0.5399 l	0.4195 m	0.4664lmn	0.3907 o
	14	0.5813 h	0.4522 j	0.4833ij	0.4239 l
	15	0.5409 l	0.4448 o	0.4748jkl	0.4001 n
	16	0.5996 f	0.4518 j	0.5214 f	0.4351 j
	17	0.5508 k	0.4602 p	0.4564 no	0.4007 n
	18	0.5842 h	0.4409 k	0.5097fg	0.4628 h
	19	0.5864 g	0.4990 f	0.5291 e	0.4709 g
	21	0.6042 f	0.5194 e	0.5326 de	0.4817 f
	22	0.5695 i	0.4771 h	0.5128fg	0.4699 g
	23	0.5914 g	0.4911 g	0.5031 g	0.4756 f
	25	0.5600 j	0.4811 h	0.4676lmn	0.4660 g
	26	0.5762 h	0.4983 f	0.5168 f	0.4745 g
	28	0.5454 k	0.4610 i	0.4918 hi	0.4589 h
	30	0.5525 k	0.4483 j	0.4639lmn	0.4287 k
	31	0.5996 f	0.4603 i	0.4995gh	0.4577 h
	33	0.5743 i	0.4521 j	0.5567 c	0.5095 e
	40	0.5684 i	0.4909 g	0.4663klm	0.3996 n
	44	0.5299 m	0.4593 i	0.4963gh	0.3830 p
	45	0.5195 n	0.4414 k	0.4863 hi	0.4776 f
	46	0.5163 n	0.4389 k	0.4780ijk	0.4673 g
	47	0.5097 o	0.4116 n	0.4685klm	0.4571 h
	48	0.5008 p	0.4629 i	0.4805ijk	0.4810 f
III	4	0.4854 r	0.3819 r	0.3668 u	0.3279 u
	6	0.4742 t	0.3778 r	0.3389 v	0.2920 x
	7	0.4951 q	0.4130 n	0.4046 r	0.3472 s
	10	0.4895 r	0.4093 n	0.4003 r	0.3428 t
	13	0.4954 p	0.4132 n	0.4072 r	0.3512 s
	20	0.4823 s	0.3921 q	0.4094 r	0.3643 r
	24	0.4697 t	0.3808 r	0.4164 q	0.3716 q

27	0.4701 t	0.3926 q	0.4307 p	0.3851 o
29	0.4175 v	0.3530 t	0.4224 q	0.3702 q
32	0.4760 s	0.3411 u	0.3921 s	0.3493 s
34	0.4770 s	0.3590 s	0.3454 v	0.3054 v
37	0.4681 t	0.3503 t	0.3803 t	0.3291 u
38	0.4736 t	0.4595 i	0.4031 r	0.3599 r
39	0.4436 u	0.3415 u	0.3711 u	0.3078 v
41	0.3814 w	0.3234 v	0.3296 w	0.3069 v
43	0.3774 w	0.3233 v	0.3663 u	0.3027 w

Group I: high *TaER* expression; Group II: intermediate *TaER* expression; Group III: low *TaER* expression. Lower case letters represent significant differences among the 48 wheat varieties ($P < 0.05$).
