# Supplementary material for: TaER Expression Is Associated with Transpiration Efficiency Traits and Yield in Bread Wheat
Source: PLoS One. 2015 Jun 5;10(6):e0128415. doi: 10.1371/journal.pone.0128415 (PMC4457575; doi:10.1371/journal.pone.0128415)
Supplement: S4 Table — (PDF) [file pone.0128415.s004.pdf]

S4 Table. *R*elative expression of three homologous copies in the A, B and D genome of *TaER1*or*TaER2* in 4 wheat varieties

**Development stage**	**Homologous genes**	**High *TaER* expression**		**Low *TaER* expression**	
		**Wheat 36**	**Wheat 42**	**Wheat 41**	**Wheat 43**
Heading (Z55)	*TaER1_AS*	0.5954	0.9906	0.4352	0.5038
	TaER1_BS	1.4046	0.9739	0.1630	0.1571
	TaER1_DS	0.5667	1.0942	0.3151	0.3021
	TaER1	**0.6977**	**0.6087**	**0.3814**	**0.3774**
	TaER2_AL	1.4290	0.7286	0.3127	0.3439
	TaER2_BL	0.8166	0.9959	0.6245	0.4525
	TaER2_DL	1.2107	1.0599	0.9153	0.8923
	TaER2	**0.5929**	**0.6183**	**0.3296**	**0.3663**
Grain-filling (Z73)	*TaER1_AS*	0.6466	0.6277	0.3076	0.3257
	TaER1_BS	0.8114	0.7440	0.4035	0.4893
	TaER1_DS	0.8918	0.5802	0.3594	0.2734
	TaER1	**0.5730**	**0.5812**	**0.3234**	**0.3233**
	TaER2_AL	0.5336	0.6067	0.2917	0.2766
	TaER2_BL	0.4411	0.6852	0.2632	0.2276
	TaER2_DL	0.5876	0.7428	0.3655	0.3289
	TaER2	**0.5845**	**0.6072**	**0.3069**	**0.3027**

No.36, 42, 41, 43 are wheat variety of Fengchan3, Drysdale, Shan7859 and Quarrion. Specific primers for *TaER1_AS*, *TaER1_BS*, *TaER1_DS*, *TaER2_AL*, *TaER2_BL* and *TaER2_DL* were used to separate the expression for each of the three copies in A, B and D genome of *TaER1* and *TaER2*. Details of these primers were listed in S2 Table.
