# Supplementary material for: TaER Expression Is Associated with Transpiration Efficiency Traits and Yield in Bread Wheat
Source: PLoS One. 2015 Jun 5;10(6):e0128415. doi: 10.1371/journal.pone.0128415 (PMC4457575; doi:10.1371/journal.pone.0128415)
Supplement: S5 Table — (PDF) [file pone.0128415.s005.pdf]

S5 Table. Transpiration efficiency related traits of 48 wheat varieties at grain- filling (Z73) stage

Group	Genotype No.	SD (No. mm^{-2})	FLA (cm^2)	A (μmol. m^{-2} s^{-1})	E (mmol. m^{-2} s^{-1})	WUEi (μmol. $mmol^{-1}$)
I	1	49.98 cd	32.18 b	21.27 bc	2.79 a	6.69 b
	3	49.64 bc	33.65 a	21.57 b	2.68 a	6.77 b
	35	48.85 a	31.12 d	21.54 b	2.92 ab	6.50 b
	36	48.73 a	31.83 c	20.92 c	2.73 a	7.20 a
	42	49.03 ab	31.32 d	22.38 a	2.70 a	6.76 b
II	2	56.21 nopq	24.19 n	18.06 ij	4.12 lmno	5.24 jklm
	5	55.89 mno	27.97 f	18.83 gh	4.17 mnop	5.34 jklm
	8	53.44 ij	24.99 l	19.68 ef	3.75 ghij	5.52 hijk
	9	54.65 l	24.09 n	17.27 mno	3.86 hijkl	5.27 jklm
	11	51.49 e	25.64 j	17.30 mno	3.90 hijklm	5.23 jklm
	12	52.34 fg	25.31 k	17.73 jkl	3.81 hijk	5.72 fghi
	14	53.03 hi	27.66 g	17.92 jk	3.93 ijklm	5.57 ghij
	15	51.34 e	25.70 j	17.84 jkl	4.11 lmno	5.90 cdefg
	16	51.66 e	28.76 e	17.82 jkl	3.69 fghi	5.73 fghi
	17	54.25 kl	23.30 p	17.11 nop	4.01 jklmno	6.02 cdef
	18	54.12 kl	27.52 gh	17.54 klmn	4.00 jklmn	5.46 ijkl
	19	52.98 ghi	27.33 h	18.74 gh	3.67 fghi	5.25 jklm
	21	53.12 hi	27.16 h	19.81 ef	3.94 ijklm	5.98 cdef
	22	55.56 mn	23.29 p	17.11 nop	3.73 ghij	6.03 cdef
	23	54.36 kl	27.78 fg	19.72 ef	3.70 fghi	5.78 defghi
	25	52.88 ghi	25.64 j	18.65 h	3.51 efg	6.16 c
	26	50.55 d	26.59 i	20.08 de	3.94 ijklm	5.84 cdefgh
	28	53.83 jk	24.93 l	18.04 ij	3.69 fghi	6.10 cde
	30	54.14 kl	24.68 m	17.64 jklm	3.47 defg	5.76 efghi
	31	52.54 fgh	26.33 i	18.45 hi	3.30 cde	5.74 efghi
	33	53.92 jk	25.36 k	17.85 jkl	3.15 bc	5.29 jklm
	40	50.16 cd	25.90 j	18.07 ij	3.63 fgh	5.48 hijkl
	44	51.48 e	24.14 n	19.51 f	3.24 cd	5.73 fghi
	45	53.96 jk	25.38 k	20.25 d	3.26 cde	5.31 jklm
	46	55.44 m	24.39 n	20.08 de	3.44 def	6.13 cd
	47	55.67 mno	24.66 m	19.76 ef	3.64 fgh	5.81 cdefghi
	48	51.94 ef	24.91 l	19.10 g	3.12 bc	5.79 defghi
III	4	60.58 v	21.69 r	16.31 r	4.76 rs	5.14 lm
	6	58.47 t	21.95 q	17.13 nop	4.83 s	4.17 o
	7	55.69 mno	22.06 q	16.51 qr	4.01 jklmno	4.98 mn
	10	56.01 mnop	17.58 x	15.13 t	4.13 lmno	5.12 lm
	13	57.22 rs	21.99 q	16.44 qr	4.54 qr	5.18 klm

20	56.08 mnop	21.62 r	16.44 qr	4.29 opq	4.99 mn
24	56.34 opq	19.64 t	16.12 rs	3.89 hijklm	5.17 klm
27	56.68 pqr	20.18 s	17.47 lmn	4.09 klmno	5.09 m
29	57.11 rs	18.09 w	16.81 pq	3.96 ijklm	5.08 m
32	57.23 rs	21.50 r	16.46 qr	4.13 lmno	4.71 n
34	60.71 v	21.57 r	17.17 nop	4.42 pq	5.02 mn
37	56.02 mnop	19.32 u	16.23 r	4.12 lmno	5.02 mn
38	56.85 qr	21.41 r	16.51 qr	4.28 nop	4.33 o
39	57.75 s	18.33 v	17.02 op	3.90 hijklm	4.98 mn
41	59.80 u	16.75 y	15.80 s	3.84 hijkl	5.09 m
43	62.09 w	16.87 y	17.66 jklm	3.88 hijklm	5.07 m

Group I: high *TaER* expression; Group II: intermediate *TaER* expression; Group III: low *TaER* expression. Lower case letters represent significant differences among the 48 wheat varieties ($P<0.05$). SD: stomatal density (No. mm^{-2}); FLA: flag leaf area (cm^2); A: photosynthetic rate (μmol. m^{-2} s^{-1}); E: transpiration rate (mmol. m^{-2} s^{-1}); WUEi: instant water use efficiency (μmol. $mmol^{-1}$).
