# Supplementary material for: TaER Expression Is Associated with Transpiration Efficiency Traits and Yield in Bread Wheat
Source: PLoS One. 2015 Jun 5;10(6):e0128415. doi: 10.1371/journal.pone.0128415 (PMC4457575; doi:10.1371/journal.pone.0128415)
Supplement: S6 Table — (PDF) [file pone.0128415.s006.pdf]

S6 Table. Carbon isotope discrimination and yield-related traits of the 48 wheat varieties

Group	Genotype No.	CID (‰)	BYPP (g)	GYPP (g)	HI
I	1	21.09 abcd	41.35 cd	18.95 bc	0.46 cdefghijk
	3	21.16 abcdef	40.82 de	18.27 c	0.45 defghijk
	35	21.11 abcde	42.42 bc	18.24 c	0.43 efghijk
	36	21.09 abcd	44.60 a	19.24 b	0.43 efghijk
	42	20.90 a	43.13 b	22.23 a	0.52 abcdefghi
II	2	21.66 hijklmno	35.93 ij	14.66 hi	0.41 efghijk
	5	21.80 jklmnopqr	35.79 ij	14.81 h	0.41 efghijk
	8	21.37 defghi	38.99 ij	15.25 gh	0.39 efghijk
	9	21.74 jklmnopq	31.31 no	16.02 efg	0.51 abcdefghi
	11	21.47 ghijk	33.31 kl	17.24 d	0.52 abcdefghi
	12	21.53 ghijk	35.99 ij	12.62 mno	0.35 hijk
	14	21.34 cdefgh	36.31 hi	15.73 fg	0.43 defghijk
	15	21.55 ghijk	33.99 kl	14.72 hi	0.43 defghijk
	16	21.45 efghij	31.21 no	17.25 d	0.55 abcdefg
	17	21.83 klmnopqr	34.56 jk	13.23 klmn	0.38 efghijk
	18	21.80 jklmnopqr	36.06 ij	14.23 hij	0.39 efghijk
	19	21.59 ghijkl	31.65 mn	16.68 de	0.53 abcdefgh
	21	21.46 fghijk	32.53 lmn	17.09 d	0.53 abcdefgh
	22	21.65 hijklmn	28.80 pqrs	14.76 hi	0.51 abcdefghi
	23	21.53 ghijk	29.11 pqr	15.97 efg	0.55 abcdefgh
	25	21.59 ghijkl	29.37 pq	15.43fgh	0.53 abcdefgh
	26	21.34 cdefgh	29.97 op	16.21 ef	0.54 abcdefgh
	28	21.56 ghijk	29.18 pqr	13.79 jk	0.47 bcdefghij
	30	21.63 ghijklm	37.70 gh	12.80 lmno	0.34 hijk
	31	21.36 defgh	38.36 fg	13.91 ijk	0.36 ghijk
	33	21.68 hijklmnop	29.94 op	12.05 opqrst	0.40 efghijk
	40	21.27 bcdefg	38.53 fg	12.23 opqr	0.32 jk
	44	21.56 ghijk	38.57 fg	12.38 nopq	0.32 ijk
	45	21.76 jklmnopqr	33.36 kl	12.16 opqr	0.36 fghijk
	46	20.94 ab	33.10 klm	12.12 opqrs	0.37 ghijk
	47	21.00 abc	31.46 no	8.21 v	0.26 k
	48	21.73 ijklmnopq	39.78 ef	11.24 stu	0.28 k
III	4	22.29 stuv	28.22 qrs	11.66 pqrstu	0.41 efghijk
	6	22.34 tuv	25.30 t	13.29 klm	0.53 abcdefgh
	7	22.04 pqrst	27.57 rs	16.24 ef	0.59 abcde
	10	22.03 pqrst	22.71 uvw	15.91 efg	0.70 a
	13	21.95 lmnopqrs	27.73 rs	10.76 u	0.39 efghijk
	20	22.07 qrstu	27.63 rs	15.80 efg	0.57 abcdef

24	22.03 pqrst	20.79 x	13.54 kl	0.65 abc
27	21.99 mnopqrst	18.59 y	12.51 mnop	0.67 ab
29	22.02 opqrst	22.45 vw	11.21 tu	0.50 bcdefghij
32	22.07 qrstu	24.12 tu	11.70 rstu	0.48 bcdefghij
34	22.40 uv	22.58 uvw	11.59 qrstu	0.51 abcdefgh
37	22.01 nopqrst	22.12 vwx	12.23 opqr	0.55 abcdefg
38	22.48 v	27.41s	15.24 gh	0.56 abcdefg
39	22.12 rstu	24.06 tu	15.24 gh	0.63 abcd
41	22.29 stuv	21.27 wx	7.84 v	0.37 efghijk
43	22.62 v	23.03 uv	8.47 v	0.37 ghijk

Group I: high *TaER* expression; Group II: intermediate *TaER* expression; Group III: low *TaER* expression. Lower case letters represent significant differences among the 48 wheat varieties (P<0.05). CID: carbon isotopic discrimination (‰); BYPP: biomass yield per plant (g); GYPP: grain yield per plant (g); HI, harvest index.
